# For avid glucose tumors, the SUV peak is the most reliable parameter for [^18^F]FDG-PET/CT quantification, regardless of acquisition time

**DOI:** 10.1186/s13550-016-0177-8

**Published:** 2016-03-05

**Authors:** Avigaëlle Sher, Franck Lacoeuille, Pacôme Fosse, Laurent Vervueren, Aurélie Cahouet-Vannier, Djamel Dabli, Francis Bouchet, Olivier Couturier

**Affiliations:** Nuclear Medicine Department, University Hospital – Angers, 4 rue Larrey 49933, Angers, Cedex 09 France; LUNAM Université – INSERM UMR-S 1066, Micro et nanomédecine biomimétiques, 4 rue Larrey 49933, Angers, Cedex 09 France

**Keywords:** [^18^F]FDG uptake, SUV_peak_, Acquisition time

## Abstract

**Background:**

This study is an assessment of the impact of acquisition times on SUV with [^18^F]FDG-PET/CT on healthy livers (reference organ with stable uptake over time) and on tumors.

**Methods:**

One hundred six [^18^F]FDG-PET/CT were acquired in list mode over a single-bed position (livers (*n* = 48) or on tumors (*n* = 58)). Six independent datasets of different durations were reconstructed (from 1.5 to 10 min). SUV_max_ (hottest voxel), SUV_peak_ (maximum average SUV within a 1-cm^3^ spherical volume), and SUV_average_ were measured within a 3-cm-diameter volume of interest (VOI) in the right lobe of the liver. For [^18^F]FDG avid tumors (SUV_max_ ≥ 5), the SUV_max_, SUV_peak_, and SUV_41%_ (isocontour threshold method) were computed.

**Results:**

For tumors, SUV_peak_ values did not vary with acquisition time. SUV_max_ displayed significant differences between 1.5- and 5–10-min reconstruction times. SUV_41%_ was the most time-dependent parameter. For the liver, the SUV_average_ was the sole parameter that did not vary over time.

**Conclusions:**

For [^18^F]FDG avid tumors, with short acquisition times, i.e., with new generations of PET systems, the SUV_peak_ may be more robust than the SUV_max_. The SUV_average_ over a 3-cm-diameter VOI in the right lobe of the liver appears to be a good method for a robust and reproducible assessment of the hepatic metabolism.

## Background

Assumed to be more accurate and less operator-dependent than visual analysis, quantification is increasingly used in positron emission tomography (PET) studies in routine practice or clinical trials. This is particularly relevant for treatment monitoring since it has been shown that objective quantification of [^18^F]FDG uptake changes may improve the prognostic value of [^18^F]FDG-PET compared with visual analysis [1]. Even prone to many sources of errors and variability, the semi-quantitative method (standardized uptake value SUV) is currently preferred to the absolute quantification of glucose metabolic rate, which requires dynamic imaging and measurement of the arterial input function, and thus considered to be too complex for a use in routine practice. SUV_max_ (SUV of the hottest voxel within a defined volume of interest (VOI)) is the most widely used parameter, easy-to-use, and operator-independent. However, SUV_max_ may be affected by noise and may merely reflect statistical fluctuations when the acquisition time is too short [2]. Among the other SUV, SUV_peak_ has been suggested as an alternative to SUV_max_ [3]. SUV_peak_ is an average SUV computed within a fixed-size VOI, most often containing (and not necessarily centered on) the hottest pixel value. Because this VOI encompasses several pixels, SUV_peak_ is assumed to be less affected by image noise than SUV_max_ [4, 5] and then more reliable and appropriate for monitoring tumor response, while remaining easy-to-use with very little or no operator dependency. The major drawback of SUV_peak_ is that its associated volume of interest (VOI_peak_) is not uniquely defined, leading to a few dozen of SUV_peak_ definitions, differing in the shape, size, and location of the VOI_peak_ [6]. In one hand, VOI_peak_ should be large enough to prevent SUV_peak_ to be affected by noise and partial-volume effects, and in other hand, VOI_peak_ should not be too large to avoid inclusion of voxels outside the tumor. These considerations lead to a fixed 1-cm^3^ sphere recommended by PERCIST [3] as a standard definition of SUV_peak_.

The use of time of flight (TOF) in reconstruction algorithms of new generations of hybrid PET/CT machines (positron emission tomography scanner/X-ray computed tomography scanner) improves signal-to-noise ratio, spatial resolution, and lesion detectability, theoretically allowing reduced injected activity (and thus radiation exposure) and/or acquisition time [7]. Furthermore, point spread function (PSF) reconstruction, also available in new generations of PET systems, is known not only to improve sensitivity but also to overestimate SUV [8, 9]. The quantitative accuracy of these techniques is not fully known [8], especially their impact on the SUV_max_ determination when acquisition time is reduced to its lower limit for optimizing acquisition protocol in clinical practice. The implementation of these new techniques therefore presents a challenge for centers to define an acquisition protocol that can be used for visual and quantitative analysis, while respecting the European Association of Nuclear Medicine (EANM) guidelines [10], i.e., either by determining the minimum FDG-administrated dose in relation to PET acquisition duration and patient weight or by choosing to apply a higher activity to reduce duration of the study. The aim of this study was to evaluate the impact of acquisition time on SUV on healthy livers (reference organs with stable uptake over time) and on tumors.

## Methods

### Materials

One hundred six whole-body PET/CT scans with 2-[^18^F]-2-deoxy-d-glucose ([^18^F]FDG) were performed in 102 patients (39 women, 63 men), for staging or for the evaluation of treatment response of neoplastic or inflammatory diseases. Patients’ characteristics and tumor histology are summarized in Table 1. The Ethics Committee of the University of Angers approved the study protocol.

**Table 1:** Patients characteristics: mean ± standard deviation

Characteristics	All	Women	Men
Age	60 ± 17		
Height (cm)	169 ± 10		
Weight (kg)	69 ± 13.2		
Body mass index (kg/m^2^)	24.2 ± 3.9	23.9 ± 4.2	24.4 ± 3.7
Injected activity (MBq)	220 ± 57		
Whole-body PET/CT uptake time (min post-injection)	62 ± 4.6		
PET/CT uptake time for 10 min LM acquisition (min post-injection)	83 ± 5.8		
Tumor histology (*n* = 58)			
Non-small cell lung cancer	29		
Mesothelioma	1		
Non-Hodgkin lymphoma including:	14		
Diffuse B cell lymphoma	6		
Follicular lymphoma	4		
T cell lymphoma	2		
Mantle cell lymphoma	2		
Hodgkin disease	6		
Multiple Myeloma	1		
Esophageal cancers	3		
Pheochromocytoma	1		
Breast cancer	1		
Cervical-uterine cancer	1		
Ovarian cancer	1		

### PET/CT scanning

Patients fasted for at least 6 h before the intravenous injection of 220 ± 57 MBq (3 MBq/kg) of [^18^F]FDG. Data were acquired on a PET/CT Discovery-690 system (LYSO scintillation PET detector; 64-slice CT; GE^®^, Buc, France) with an acquisition time of 3 min/bed position. PET images were reconstructed with an ordered-subset expectation maximization (OSEM) 3D algorithm (3 iterations, 8 subsets, 192 × 192 matrix, 3.65 mm pixels, slice thickness 3.27 mm, post-reconstruction Gaussian 4 mm filter) with VPFX time-of-flight (TOF) algorithm and point spread function (PSF) correction (Sharp IR). CT-based attenuation correction (120 kV, Auto mA, collimation 20 mm, pitch 1.375, 0.8 s/rot) was applied.

Whole-body PET/CT were performed 62 ± 4.6 min after [^18^F]FDG injection, and an additional PET/CT acquisition of 10 min in list mode (LM) was acquired using a single-bed position 83 ± 5.8 min after [^18^F]FDG injection.

After the whole-body scan acquisitions (*n* = 106), additional list mode acquisitions were performed on the most avid tumor (tumor SUV_max_ > 5; *n* = 58) or on normal healthy liver (no history of liver metastasis and no evidence of liver lesion on whole-body [^18^F]FDG-PET/CT scans; *n* = 48).

Six datasets (called R for replay) were reconstructed from this LM additional acquisition, mimicking acquisition times of 1.5, 2, 2.5, 3, 5, and 10 min (respectively R_1.5,_ R_2_, R_2.5_, R_3_, R_5_, and R_10_).

### Image analysis

Two experienced nuclear medicine physicians analyzed all images datasets on an Imagys^®^ workstation (Keosys^®^, Saint-Herblain, France), allowing the computation of different SUV. SUV was corrected for body weight (SUVbw).

For tumor [^18^F]FDG uptake quantification, a manual VOI encompassing the entire tumor was drawn on R_10_. VOIs were registered and repositioned identically for the five other replays (R_1.5_ to R_5_) using 3D coordinates that allow the reposition of the VOI on all replays. SUV_max_ (SUV of the hottest voxel), SUV_peak_ (maximum average SUV within a 1-cm^3^ sphere), SUV_41%_ (threshold-based tumor delineation applying a threshold of 41 % of the SUV_max_), and metabolic tumor volume (applying a threshold of 41 % of the SUV_max_ on R_10_) were then automatically generated. A threshold of 41 % was chosen following the EANM guidelines [10] and because the most common thresholding value chosen in the clinical setting is 40–43 % of the SUV_max_.

For the liver, a 14-cm^3^ VOI was positioned on the right lobe of the liver on R_10_, as proposed in PERCIST [3]. As for tumors, VOIs were repositioned identically for the five other replays using 3D coordinates that allow the reposition of the VOI on all replays. SUV_max_, SUV_peak_, and SUV_average_ (average SUV within the fixed 14-cm^3^ VOI) were automatically generated.

### Statistical analysis

For tumors, SUV_max_, SUV_peak_, and SUV_41%_ were analyzed. For livers, SUV_max_, SUV_peak_, and SUV_average_ were analyzed.

Paired *t* tests were performed to study the inter-observer reproducibility of tumor and liver measurements.

Repeated-measures ANOVA (Tukey post-tests) was performed to test the variations of measurements over time, i.e., between the six replays (R_1.5_ to R_10_).

Individual SUV fluctuations ∆t over time (i.e., at 1.5, 2, 2.5, 3, and 5 min) were evaluated using the SUV at 10 min as reference SUV and were calculated as follows:

$$ {\varDelta}_t=\frac{\left({\mathrm{SUV}}_t-{\mathrm{SUV}}_{10 \min}\right)}{{\mathrm{SUV}}_{10 \min }} $$

For each replay, and for liver and tumors, the maximum individual fluctuations were registered.

Statistical tests were performed using Prism 4 software (GraphPad software, CA, USA). The level of significance was set at 5 %.

## Results

No significant differences were observed between the two readers for the evaluated image datasets (tumor and liver).

### Tumors

SUV_peak_ was the only parameter stable over time with no significant statistical difference between the six replays.

SUV_max_ and SUV_41%_ decreased significantly with time (*p* ≤ 0.0005) (Table 2). For SUV_max_, statistical differences were observed between the shortest acquisition time R_1.5_ versus the longest R_5_ and R_10_ (*p* = 0.0005).

**Table 2:** SUV_max_, SUV_41%_/SUV_average_, and SUV_peak_ (mean ± SD of all datasets) of tumors and livers for each replay

	SUV R_1.5_ (mean ± DS)	SUV R_2_ (mean ± DS)	SUV R_2.5_ (mean ± DS)	SUV R_3_ (mean ± DS)	SUV R_5_ (mean ± DS)	SUV R_10_ (mean ± DS)	*p* ANOVA
Tumor							
SUV_max_	17.4 ± 9.5 (R_5_, R_10_)	17.3 ± 9.6	17.2 ± 9.7	17.1 ± 9.6	16.9 ± 9.5	16.9 ± 9.7	0.0005
SUV_41%_	10.4 ± 5.6 (R_5_, R_10_)	10.3 ± 5.5 (R_5_, R_10_)	10.2 ± 5.6 (R_10_)	10.2 ± 5.6	10.1 ± 5.5	10.1 ± 5.6	<0.0001
SUV_peak_	13.6 ± 8.3	13.6 ± 8	13.6 ± 8.1	13.5 ± 8	13.6 ± 8.1	13.5 ± 8.2	NS
Liver							
SUV_max_	2.9 ± 0.5 (R_2.5_ → R_10_)	2.8 ± 0.5 (R_3_ → R_10_)	2.8 ± 0.5 (R_5_, R_10_)	2.7 ± 0.5 (R_5_, R_10_)	2.6 ± 0.5 (R_10_)	2.5 ± 0.5	<0.0001
SUV_average_	2.2 ± 0.4	2.2 ± 0.4	2.2 ± 0.4	2.2 ± 0.4	2.2 ± 0.4	2.2 ± 0.4	NS
SUV_peak_	2.5 ± 0.4 (R_2_ → R_10_)	2.5 ± 0.4 (R_5_, R_10_)	2.4 ± 0.4 (R_5_, R_10_)	2.4 ± 0.4 (R_5_, R_10_)	2.4 ± 0.4	2.4 ± 0.4	<0.0001

(R_2_) means a significant difference with the replay R_2_, (R_2.5_) with R_2.5_, (R_3_) with R_3_, (R_5_) with R_5_, and (R_10_) with R_10_

SUV_41%_ was the most time-dependent parameter, with significant statistical differences between R_1.5_ and R_2_ versus R_5_ and R_10_ and between R_2.5_ and R_10_ (*p* < 0.0001) (Table 2).

SUV_peak_ was the least variable parameter with individual fluctuations up to 38 % (from 7.22 to 9.96) versus 58 % for SUV_max_ (from 10.82 to 17.1) and 56 % for SUV_41%_ (from 6.22 to 9.7) (Table 3). For all tumor SUVs, maximal fluctuations were observed between the shortest replay (R_1.5_ or R_2_) and R_10_. Considering a maximum fluctuation of 10 % as an acceptable level of variation for tumor SUV [11], the number of patients with tumor SUV fluctuations >10 % (compared to R_10_) was noted. R_1.5_ and R_2_ were the replays in which the higher number of patients with SUV fluctuations >10 % was observed, whatever the type of SUV. SUV_peak_ was the least variable parameter with fluctuations >10 % observed in only five patients (R_1.5_) compared to SUV_max_ (11 patients, R_1.5_) and SUV_41%_ (seven patients, R_2_).

**Table 3:** Maximal individual SUV fluctuations (Δmax) compared to the reference SUV at 10 min

	Δmax R_1.5_ vs R_10_ ( %)	Δmax R_2_ vs R_10_ ( %)	Δmax R_2.5_ vs R_10_ ( %)	Δmax R_3_ vs R_10_ ( %)	Δmax R_5_ vs R_10_ ( %)	Δmax mean value ( %)
Tumor						
SUV_max_	58	43	31	24	31	37
SUV_41%_	56	41	26	25	26	35
SUV_peak_	34	38	29	26	13	28
Liver						
SUV_max_	41	35	26	29	19	30
SUV_average_	16	15	14	14	9	14
SUV_peak_	17	19	19	18	11	17

Δmax R_t_-R_10_ (%) is the maximal individual fluctuation between SUV value at R_t_ and SUV value at R_10_. Δmax mean value is the mean Δmax from “R_1.5_ vs R_10_” to “R_5_ vs R_10_”

Metabolic tumor volumes (VOI_41%_) measured on R_10_ varied widely from 1.1 to 369.32 cm^3^ (median 6.47 cm^3^).

### Livers

SUV_average_ was stable over time with no significant statistical difference, whereas a significant tendency to decrease with time was observed for SUV_max_ and SUV_peak_ (*p* ≤ 0.0005; Table 2).

For SUVpeak, statistical differences were observed between R_1.5_ and the other replays and between R_2_, R_2.5_, and R_3_ versus R_5_ and R_10_ (*p* < 0.0001).

SUV_max_ was the most time-dependent parameter (*p* < 0.0001) (Table 2).

SUV_average_ was the least variable parameter with individual variations up to 16 % (between R_1.5_ and R_10_) versus 19 % for SUV_peak_ and 41 % for SUV_max_ (between R_1.5_ and R_10_).

## Discussion

The SUV definitions used in the present study are usually described to be particularly suitable for response-monitoring purposes. Indeed, these semi-automatic and operator-independent methods allow a simple and reproducible evaluation of SUV that is a basic requirement to provide an added value to the quantitative measurement compared to visual assessment. There is no actual consensus about the best SUV parameter to be used to assess response to therapy, but most response assessment studies use SUV_max_, the first reason being related to its easy implementation and operator-independent character. Moreover, for small lesions or during an effective treatment with a decrease in tumor size, when partial-volume effect may result in an underestimation of [^18^F]FDG uptake, SUV_max_ may be best suited as metabolic index than the other SUVs [12]. In a clinical point of view, identification of a suitable SUV for response quantification requires clinical trials with patients’ clinical outcomes, and SUV_max_ demonstrated positive predictive values and accuracies for outcome prediction in lymphoma [1] as well as in solid tumors [13, 14]. Although the increase in counting statistics with new PET/CT systems contributes to reduce image noise, SUV_max_ remains adversely affected by noise, leading to uncertainty in the uptake quantification and thus in the treatment response categorization. Theses inaccuracies may be more pronounced in case of tumor heterogeneity that moreover may change during the course of treatment [15]. Consequently, SUV_peak_ has been recently proposed as a more robust alternative of SUV_max_ [3]. SUV_peak_ has a larger volume compared to the single pixel value of SUV_max_ and thus should be less affected by image noise [4, 5, 16].

Acquisition times recommended by the manufacturer are most often used in clinical routine, even though the actual influence of acquisition time upon SUV is not fully known. Regarding different quantifications of the same metabolic process (for a given tumor), a correlation does exist between SUV_max_ and SUV_peak_ and the uncertainties of these two parameters are probably comparable, although from different causes [4]. Nevertheless, these considerations do not allow an assessment of the influence of acquisition time on SUV, and substantial differences may exist between SUV_max_ and SUV_peak_ in individual tumors that affect the treatment response quantification and the response categorization.

Because the current worldwide trend is to reduce patient exposure to ionizing radiation, it is not conceivable to increase the injected activity to overcome a low statistical quality of PET images due to a too short acquisition time. Conversely, increasing the acquisition time may lead to discomfort of the patient and to motion artifacts. In our study, the highest fluctuations of SUV were observed when acquisition times were the shortest, whereas no significant difference was observed for replays superior or equal to 3 min. Brown et al. [17] investigated the effect of varying acquisition times on phantom and patient PET images on a 3D GE Discovery-STE PET/CT system. Patient data were investigated using list mode acquisition to obtain comparable 2-, 3-, and 4-min frames. As we reported for tumors, no significant difference was observed over 3 min at standard clinical [^18^F]FDG activities. In two other studies, the image quality was slightly adversely affected by an acquisition time of 1.5 min compared to 3 min [18] and the volume and SUV variability were significantly larger for images with scan times below 3 min [19].

For tumors, we did not observe any significant difference for SUV_peak_ over time, and regarding individual variations, SUV_peak_ was also the least variable parameter. Using a reference time of 15 min, Lodge et al. [20] reported similar results with a significant lower bias for the SUV_peak_ compared to the SUV_max_ for the 1-, 2-, 3-, and 4-min images. In our study, large SUV_max_ variations up to 58 % (R_1.5_ versus R_10_) were observed for the same tumor in the same patient, which is obviously unacceptable for response-monitoring purposes, particularly when accumulated to other sources of bias [11, 21] and if a threshold value is applied to determine treatment response as with PERCIST criteria [3]. As previously reported [2, 4, 22], we noted that fluctuations of SUV_max_ also affected threshold method SUV since the VOI was determined by selecting pixel values equal to 41 % of the maximum pixel value.

For all these reasons discussed, SUV_peak_ may be a robust alternative for the assessment of [^18^F]FDG avid tumor uptake at standard clinical activities. We implemented a SUV_peak_ algorithm using a fixed-size 1-cm^3^ VOI, automatically positioned so as to maximize the enclosed average (maximum average), typically including but not necessarily centered on the maximum pixel value. However, for small tumor sizes, the automatic placement of the VOI may be impossible, but in these cases, the placement of the VOI can be performed manually.

Finally, an important additional result is the absence of significant difference for the liver SUVaverage over time. These results confirm that the liver metabolism can be used as the reference organ for quality comparison of repeated [^18^F]FDG-PET studies in the same patient, or as a reference threshold for evaluating tumor response. In PERCIST criteria, the size of the VOI and its placement in the right lobe are mentioned, but not the position to which the measure should be made. However, a recent study found an excellent inter-observer agreement and no significant difference whether the VOI is placed in the upper part, the portal level, or at the bottom of the right liver lobe [23]. Our results are also in agreement with those of Grohien et al. [24], who showed a larger dispersion of values and a higher variance for hepatic SUV_max_ compared to hepatic SUV_average_.

## Conclusions

Tumor SUV_peak_ (volume 1 cm^3^) was the most stable quantitative parameter for acquisition times over 1.5 min at standard clinical [^18^F]FDG activities.

Although not yet widely available, commercial development of SUV_peak_ may increase the reproducibility and accuracy of quantitative PET studies, this quality of measure being essential for response-monitoring purposes. Average SUV over a 3-cm VOI in the right liver lobe was a good method for a robust assessment of hepatic metabolism, confirming the choice of the liver as the reference organ in [^18^F]FDG studies.
